# Trait related aberrant connectivity in clinically stable patients with schizophrenia: A seed based resting state fMRI study

**DOI:** 10.1007/s11682-022-00731-9

**Published:** 2022-10-14

**Authors:** Paris Alexandros Lalousis, Aanya Malaviya, Rachel Upthegrove, Kareen Heinze, Ana Diukova, Dorothee Auer, Peter Liddle, Pavan Mallikarjun

**Affiliations:** 1 Institute for Mental Health, University of Birmingham, 52 Pritchatts Road, Birmingham, B15 2SA UK; 2 Centre for Human Brain Health, University of Birmingham, Birmingham, B15 2SA UK; 3 School of Medicine, University of Nottingham, Nottingham, NG7 2RD UK

**Keywords:** Schizophrenia, Functional connectivity, Dysconnectivity, Lingual gyrus, DPARSFA

## Abstract

Aberrant resting-state connectivity within and between the Default Mode Network, the Executive Control Network, and the Salience Network is well-established in schizophrenia. Meta-analyses have identified that bilateral lingual gyrus is as the only region showing hyperactivity in schizophrenia and there are reports of increased connectivity between the lingual gyrus and other brain regions in schizophrenia. It is not clear whether these abnormalities represent state or trait markers of the illness, i.e., if they are only present during the acute phase of the illness (state) or if they reflect a predisposition to schizophrenia (trait). In this study, we used a seed-based functional connectivity analysis to investigate brain networks in schizophrenia patients who are in the stable phase of their illness and assess functional connectivity using seeds in the lingual gyrus, the posterior cingulate, the right dorsolateral prefrontal cortex (dlPFC), the right anterior insula (rAI) and the right orbital frontoinsula. Twenty patients with schizophrenia in a stable phase of their illness (as defined by the course of illness and Signs and Symptoms of Psychotic Illness (SSPI) scores) and 20 age and sex-matched healthy controls underwent resting-state functional Magnetic Resonance Imaging (rs-fMRI). Data was analysed using the Data Processing Assistant for Resting-State fMRI Advanced Edition (DPARSFA) V3.1 (http://rfmri.org/DPARSF) and the statistical parametric mapping software 8 (SPM8). Compared with healthy controls, patients with schizophrenia showed increased connectivity between the left lingual gyrus and the middle frontal gyrus, and the cingulate cortex. Lingual gyrus hyper-connectivity may be a stable trait neuroimaging marker for schizophrenia. Our findings suggest that aberrant connectivity in major resting-state networks may not be present after the acute illness has stabilised.

**Supplementary Information:**

The online version contains supplementary material available at 10.1007/s11682-022-00731-9.

## Introduction

The neurodevelopmental model of schizophrenia postulates that schizophrenia results from genetically and environmentally determined abnormal neural development from conception (Murray & Lewis, 1987). Central to this model, the dysconnectivity hypothesis proposes that functional brain networks are abnormally integrated and attempts to provide explanatory links between the symptomatology of schizophrenia and underlying neural correlates (Friston et al., 2016). While functional dysconnectivity in schizophrenia has provided biological evidence for core schizophrenia symptomatology, including positive symptoms of hallucinations and delusions (Giraldo-Chica & Woodward, 2016; Mallikarjun et al., 2018; Skåtun et al., 2018), finding differential biological “trait” instead of “state” markers of schizophrenia has remained elusive (Chen et al., 2006). Trait markers represent the features of the mechanisms that may have a causal role in the development of pathophysiology over time, whereas state markers demonstrate the real-time status of clinical manifestations of a psychiatric disorder or syndrome (Chen et al., 2006).

Resting-state functional Magnetic Resonance Imaging (rs-fMRI) is a helpful tool for assessment of dysconnectivity (Karbasforoushan & Woodward, 2013). Research in brain connectivity in chronic and partially remitted schizophrenia has shown that the core symptoms of the disorder are related to dysconnectivity between brain regions identified in networks such as the Default Mode Network (DMN), the Executive Control Network (ECN), and the Salience Network (SN) ((Kasparek et al., 2013; Manoliu et al., 2013; Woodward et al., 2011)). These brain regions include the middle frontal gyrus, the medial frontal gyrus, the superior temporal gyrus, the dorsolateral prefrontal cortex (dlPFC), the anterior cingulate gyrus, the thalamus, the cerebellum, the insula, and the claustrum (Mallikarjun et al., 2018; Pettersson-Yeo et al., 2011).

While these major functional connectivity networks have been extensively examined (Woodward et al., 2011), findings have been contradictory, with some studies reporting high connectivity between the regions that form the DMN (Whitfield-Gabrieli et al., 2009; Zhou et al., 2007), while others reported low connectivity (Bluhm et al., 2007; Rotarska-Jagiela et al., 2010). Nevertheless, most functional connectivity studies in schizophrenia have demonstrated that brain networks in schizophrenia are aberrantly connected (regardless of whether this aberration is characterised by hyper- or hypo- connectivity).

Connectivity within the DMN and between the DMN and other networks, and between the cerebellum and other cerebral regions, have been proposed as a trait pattern that may reflect a possible neuroendophenotype for the development of schizophrenia (Peeters et al., 2015; Yu et al., 2013). However, similar DMN and cerebellar dysfunctional connectivity patterns have been suggested to provide insights into the pathophysiological mechanisms of schizophrenia rather than represent trait markers (Yu et al., 2013; Mikolas et al., 2016). Furthermore, aberrant connectivity between the anterior insula and orbitofrontal areas has been suggested to reflect a trait mechanism (Mikolas et al., 2016). When examining resting-state connectivity in schizophrenia, the psychotic phase of the illness does not allow for the elucidation of whether aberrant connectivity plays a trait or state role (Manoliu et al., 2013). Studies in patients with schizophrenia who are in remission have shown that resting-state network dysfunction is still present in psychotic remission and may, therefore, reflect a trait mechanism (Kasparek et al., 2013; Manoliu et al., 2013). Whitfield-Gabrieli et al., based on their study of first-degree relatives of patients with schizophrenia, have suggested that resting-state hyperconnectivity rather than reductions in connectivity may have a genetic basis and reflect a trait biomarker for the development of schizophrenia (Whitfield-Gabrieli et al., 2009). A meta-analysis by Kuhn and Galinat, that examined measures of local neural activity and connectivity, concluded that the lingual gyrus was the only region in which patients with schizophrenia exhibited increased activity compared with controls (Kuhn & Gallinat, 2013). Furthermore, Palaniyappan and Liddle demonstrated, in a sample of similarly stable phases of illness, that patients with schizophrenia exhibited higher connectivity between the lingual gyrus and other brain regions (Palaniyappan & Liddle, 2014).

Thus, it is not yet clear whether functional dysconnectivity in schizophrenia is endophenotypic of the disorder’s underlying biology or the result of the onset and severity of symptoms. This study aimed to examine the differences in brain connectivity between patients with schizophrenia who are in the stable phase of their illness and healthy controls. We hypothesise that functional dysconnectivity is a trait marker of schizophrenia and that the areas of the brain linked to the DMN, the ECN, the SN, and the lingual gyrus would show aberrant resting-state connectivity compared to controls.

## Materials and methods

### Subjects

We recruited 20 patients with psychosis (ages 21–46) and 20 matched healthy controls (ages 19–46). Patients were recruited from the Nottinghamshire Healthcare NHS Trust. Two patients did not complete the study, and one patient and one healthy control were excluded due to excessive head movements in the scanner. The inclusion criteria for the patient group of this study were: 1) a diagnosis of schizophrenia or schizoaffective disorder as per Diagnostic and Statistical Manual IV (American Psychiatric Association, 1994) criteria established based on a clinical interview, reference to case files and a clinical consensus diagnostic meeting of research psychiatrists, 2) age between 18 and 50, and 3)in a stable phase of the illness (defined as having had no change in global severity of illness greater than 10 units out of a total range of 100 units), assessed using the Global Assessment of Functioning scale in the 6 weeks preceding the study. The exclusion criteria were: 1) a history of severe head trauma, major neurological disorder or somatic disorder with neurological complaints (e.g. multiple sclerosis), learning disability or current major medical illness, 2) current psychoactive substance use or current or previous substance dependence as defined by DSM IV) 3) any current psychiatric diagnoses other than psychosis, and 4) unsuitability to undergo MRI scan, as measured by the MRI safety-screening questionnaire. The exclusion criteria for the healthy control group of this study were: 1) a history of head trauma, major neurological disorder or somatic disorder with neurological complaints (e.g. multiple sclerosis), learning disability, or current major medical illness, 2) current psychoactive substance harmful use or current or previous substance dependence as defined by DSM IV 3) personal or family history of psychotic disorder, and 4) unsuitability to undergo MRI scan, as measured by the MRI safety-screening questionnaire. Informed consent was obtained for all of the subjects. More information about the sample demographics can be found in Table 1. All the participants answered the MRI safety‑screening questionnaire developed by the Sir Peter Mansfield Magnetic Resonance Imaging Centre to ensure that they are safe to have an MRI. The Annett Handedness scale (Annett, 1970), the Quick Test for current intellectual functioning (Ammons & Ammons, 1962), the Signs and Symptoms of Psychotic Illness (SSPI) questionnaire (Liddle et al., 2002), and the NS-SEC—National Statistics Socio-economic Classification. The GAF was used to determine stability in the phase of the illness. The GAF measures global severity of illness and consists of a 1–90 scale in 10-point intervals (American Psychiatric Association, 1994).

**Table 1:** Social and clinical demographics of the sample

	Patients (*n* = 17)	Healthy controls (*n* = 19)	Test statistic	*P* Value
Age (Mean ± S.D.)	30.41 ± 6.78	31.16 ± 6.71	*t* = .331	*p* = .743
Gender (Male/Female)	12/5	13/6	*χ*^*2*^ = .932	*p* = .628
GAF Symptom Severity (Mean ± S.D.) GAF Level of Functioning (Mean ± S.D.)	73.13 ± 13.15 73.44 ± 13.50			
Duration of Illness (Mean ± S.D.)	6.23 ± 5.21			
Age of Onset (Mean ± S.D.)	24.38 ± 5.96			
Course of Illness (Remitting/Episodic/Continuous)	10/4/3			
Medicated/Non-Medicated	14/3			
CPZ Equivalents (Mean mg ± S.D.)	364 ± 3.57			
Type of Medication (Olanzapine/Clozapine/Risperidone/ Aripiprazole)	4/3/4/3			
SSPI (Mean ± S.D.)	8.88 ± 6.95			

### Procedure

#### Paradigm and study design

Whole-brain fMRI recordings were made while the participants were lying down quietly with their eyes closed during the five minutes of resting state. They were instructed to try not to move (eyes, head, arms, etc.), try to be relaxed as much as possible and try to clear their mind of any thoughts while undergoing the scan.

#### FMRI data acquisition

Imaging was performed at a Philips Achieva 3 Tesla MRI scanner equipped with the 8-channel SENSE head coil. Thirty-five functional slices were obtained parallel to the anterior commissure–posterior commissure line, using a gradient-echo echo-planar imaging (EPI) sequence with an echo time of 35 ms, a flip angle of 90°, a repetition time (TR) of 2100 ms, flip angle of 90°, matrix 64 × 64, voxel size 3.25 × 3.25x3 mm, 35 slices and the slices were contiguous (zero slice gap). A total of 136 volumes were acquired using interleaved acquisition.

### Data analysis

#### Demographic and clinical data analysis

Age and gender were compared between the healthy control and the patient group using t-tests and chi-square tests. A Pearson Correlation Coefficient test between the statistical significant connectivity results and CPZE equivalent medication was performed to investigate potential medication effects. Means and standard deviations were calculated for all the measures used in the study.

#### FMRI data analysis

##### Preprocessing

Data Processing Assistant for Resting-State fMRI Advanced Edition (DPARSFA) V3.1 (http://rfmri.org/DPARSF) (Yan & Zang, 2010) was used to pre-process and analyse the data. The first four volumes from each scan were excluded as dummy scans to allow the stability of longitudinal magnetisation. The images were then reoriented. Image acquisition time between slices for the remaining 132 volumes was corrected using slice timing, with the middle slice being the reference slice. Subject movements in the scanner were corrected for using a six-parameter rigid-body transform that contained three translations and three rotations in and about each of the axes (x, y, and z). Two subjects were removed from the subsequent analysis due to excessive head motion (translation > 2.0 mm and rotation > 2.0°). Voxel specific motion correction (Satterthwaite et al., 2013) was also applied to ensure the quality of our images. Both the T1 and the functional images were reoriented to improve coregistration accuracy. Brain extraction was then performed on the T1 images to remove the skull before coregistration to the functional images and improve the coregistration algorithm. The T1 images were then coregistered to the functional images. The images were then segmented into gray matter (GM), white matter (WM), and cerebrospinal fluid (CSF) partitions. The Friston 24-parameter model (Friston et al., 1996) was then used to obtain 6 head motion parameters, 6 head motion parameters one time-point before, the 12 corresponding squared items and input them into our model as nuisance regressors. Any time-points that had a framewise displacement (Power et al., 2012) of more than 0.5 as well as one time-point before them and two time-points after them were regressed out. The WM and the CSF masks created during segmentation were then combined, and their time series was calculated. Their first five principal components were then calculated and entered as nuisance regressors in the model (CompCor) ( (Behzadi et al., 2007)). Diffeomorphic Anatomical Registration using Exponentiated Algebra (DARTEL) ((Ashburner, 2007)) was used to create a group-specific template to which all images were normalised. An affine transformation of the images to the Montreal Neurological Institute (MNI) stereotactic space using the parameters estimated in DARTEL was performed. Finally, the images were smoothed with a Gaussian kernel of 8 mm Full-Width at Half Maximum (FWHM).

##### Functional connectivity analysis

A seed-based correlation method was used in the healthy controls and patients to examine resting-state functional connectivity within subjects’ brains. Considering the literature mentioned above, five spherical ROIs with a 6 mm radius were chosen for this study. The ROIs and their MNI coordinates can be viewed in Table 2. For the DMN, the Posterior Cingulate was used as a seed. For the ECN, the Right dlPFC was used, and for the SN, the rAI and the right Orbital Frontoinsula were used. The lingual gyrus seed coordinates can be found in Table 2. Using Data Processing Assistant for Resting-State fMRI Advanced Edition (DPARSFA) V3.1 (http://rfmri.org/DPARSF) (Yan & Zang, 2010) an image that calculated the voxel-wise temporal correlation between the individual time series of each seed ROI and the time series of every other voxel in the brain was generated.

**Table 2:** ROIs for functional connectivity analysis (MNI coordinates)

Brain Area	X Axis	Y Axis	Z Axis
Right Anterior Insula (SN) (Palaniyappan and Liddle 2014)	33	21	–3
Right Orbital Frontoinsula (SN) (Seeley et al. 2007)	38	26	–10
Posterior Cingulate (DMN) Kuhn and Gallinat 2013)	–1	–29	26
Right dorsolateral PFC (dlPFC) (ECN) (Seeley et al. 2007)	44	36	20
Left Lingual Gyrus (Kuhn and Gallinat 2013)	–11	–57	2
Right Lingual Gyrus (Kuhn and Gallinat 2013)	11	–55	2

##### Group analysis

Group analyses were conducted using SPM8’s second level model specification. After calculating the temporal correlation between the time series of each seed ROI and every other voxel in the brain with DPARSFA, each subject had a z-score map for each ROI. For each ROI, we grouped all the z-scores of the healthy controls, and all the z-scores of the patients together. The comparison was conducted using a two-sample t-test that tested the differences between the contrast images of the two groups at p < 0.05 FDR-corrected at the cluster level. A power analysis was undertaken to test the achieved power needed for this comparison. Details can be found in the supplement (1.1).

## Results

### Demographic and clinical data

Demographic and clinical data are summarised in Table 1. All patients had a diagnosis of paranoid schizophrenia. The mean dose of antipsychotic medication in chlorpromazine equivalents was 364 mg (*SD* = 3.57). Three patients were receiving no antipsychotic medication at the time of the study. The mean duration of illness was 6.23 years. The mean SSPI score was 8.88.

### Seed-based functional connectivity analysis

**Fig. 1:**
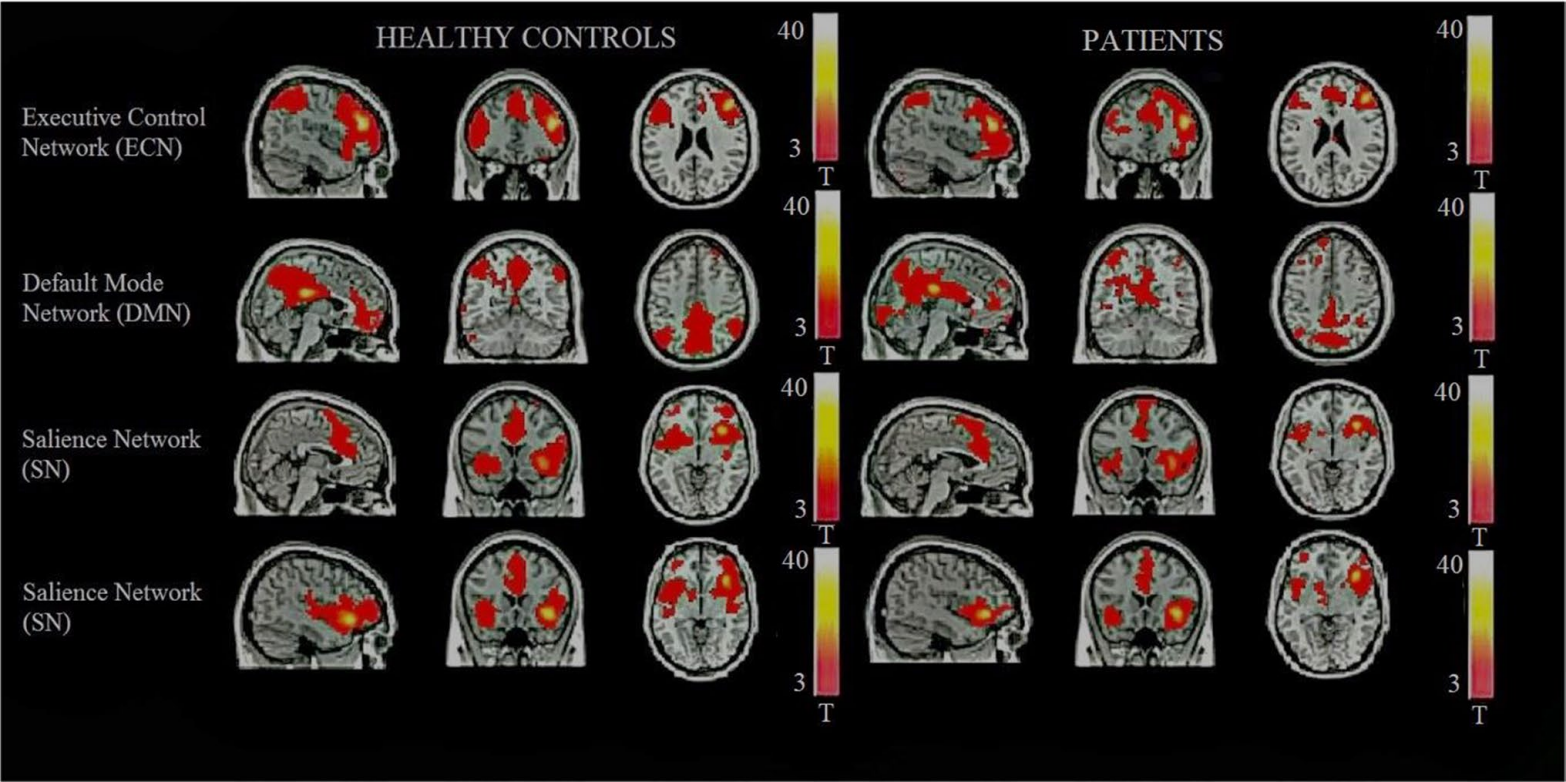
Resting state networks in healthy controls and patients exhibited using a one sample t-test. Row 3 shows the right Anterior Insula seed by Palaniyappan et al. 2014 and row 4 shows the Right Orbital Frontoinsula seed by Seeley et al. 2007

**Fig. 2:**
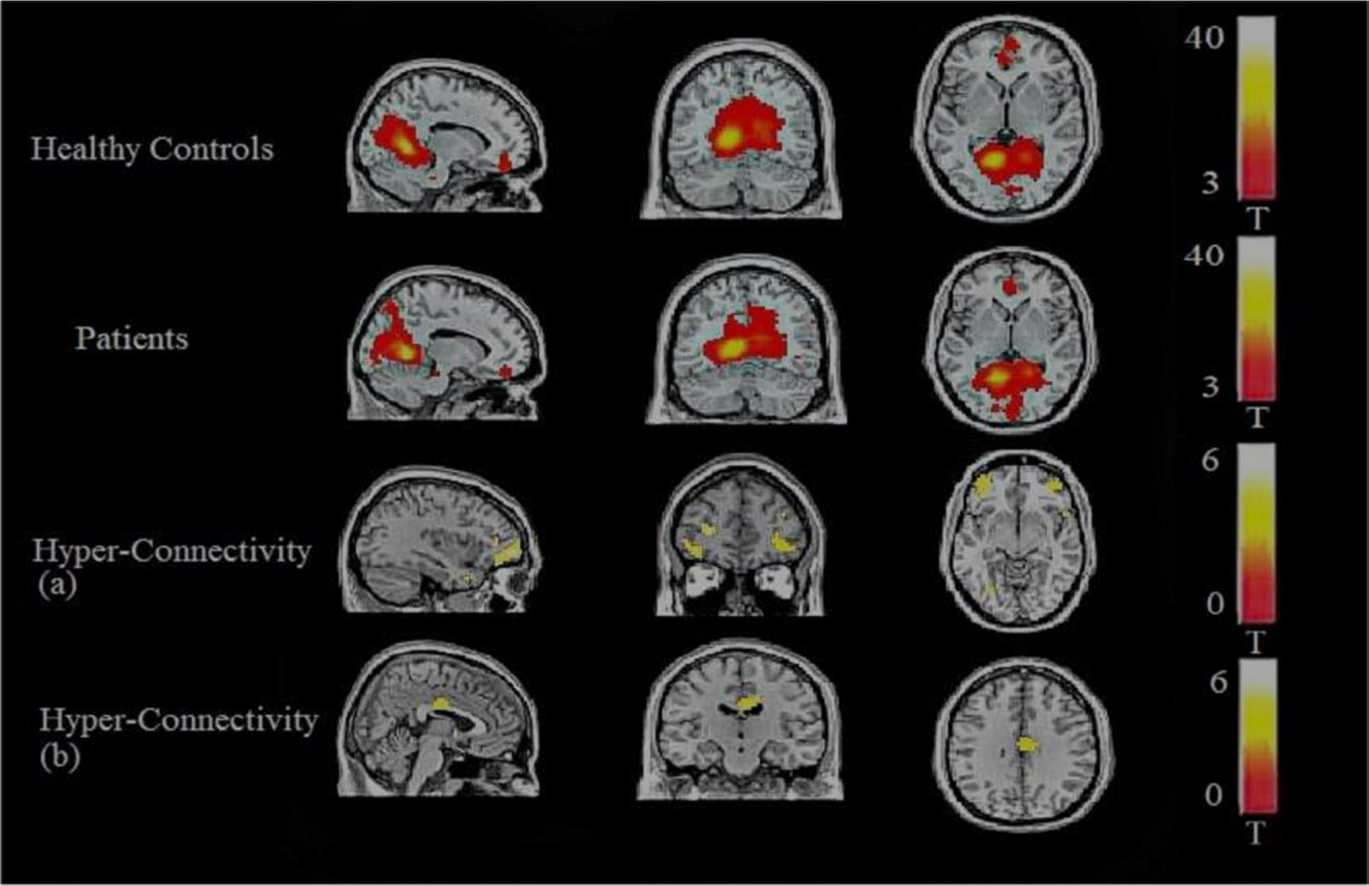
Lingual Gyrus Functional Connectivity. Comparison between Healthy Controls and Patients. Hyperconnectivity between the left lingual gyrus and the middle frontal gyrus (a) and the left lingual gyrus and the cingulate gyrus (b)

**Fig. 3:**
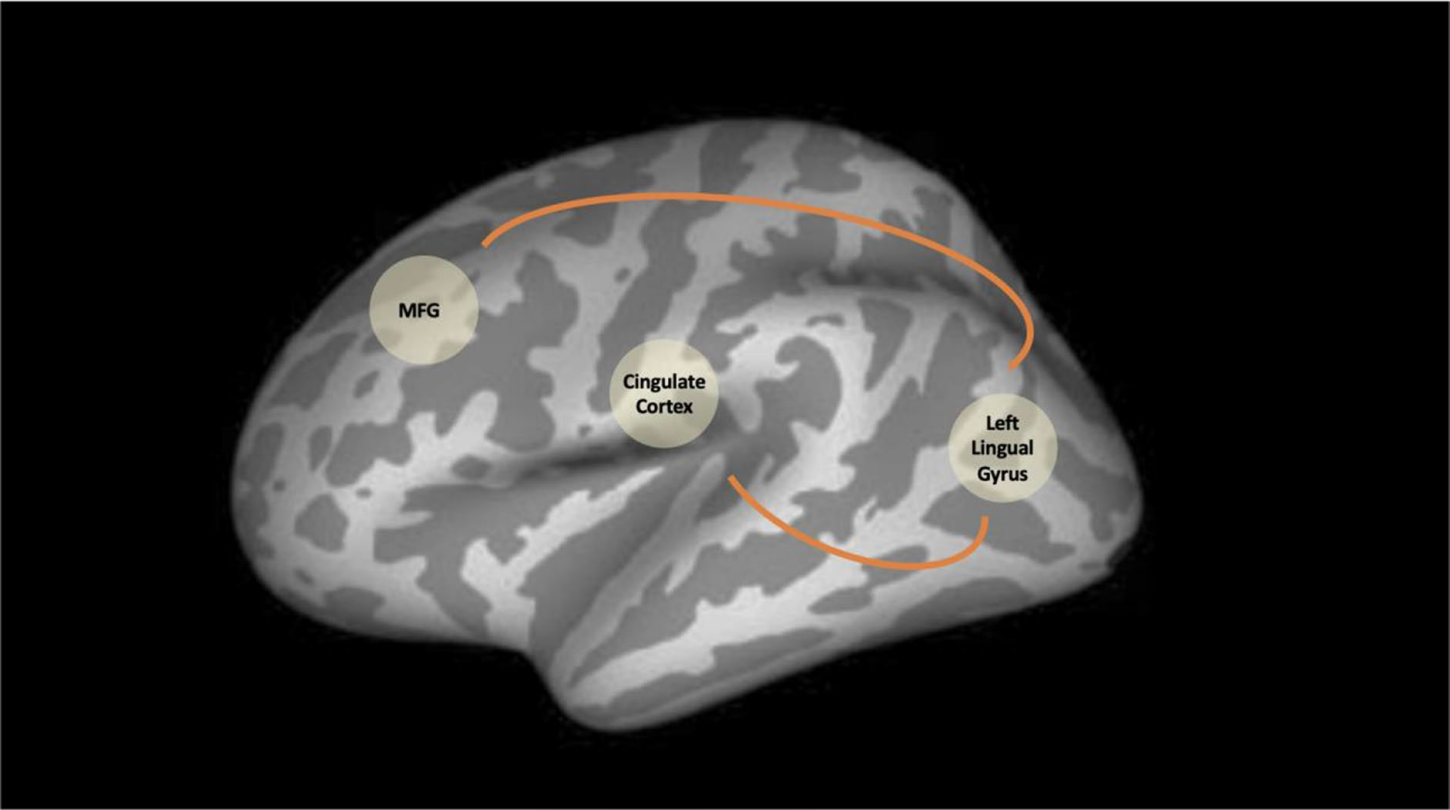
Statistically significant increased levels of seed-based functional connectivity between the left lingual gyrus and the MFG (middle frontal gyrus) and between the left lingual gyrus and the cingulate cortex in patients with schizophrenia compared to healthy individuals. The orange arrow demonstrates the direction of the connectivity

Five regions of interest (ROI) were used as seeds for the functional connectivity analysis. The ROI seeds are summarised in Table 2. There were statistically significant differences between patients and healthy controls in left lingual gyrus connectivity. Patients with schizophrenia revealed higher functional connectivity between the left lingual gyrus and the middle frontal gyrus and between the left lingual gyrus and the cingulate cortex when compared to controls (Table 3, Figs. 1,2, and 3). The brain regions linked to the DMN, the ECN, and the SN appeared to have hypoconnectivity in schizophrenia patients compared to healthy controls. However, these differences were not statistically significant. These differences are shown in Fig. 1. The functional connectivity results between the left lingual gyrus and the middle frontal gyrus and CPZE equivalent medication (r = -0.100, p = 0.723) and the left lingual gyrus and the cingulate cortex and CPZE equivalent medication (r = 0.087, p = 0.757) were not significantly correlated.

**Table 3:** Functional connectivity analysis results

Patients-healthy controls	Seed MNI coordinates	Connecting cluster peak Voxel MNI coordinates	*P* Value, peak intensity, cluster size
Left Lingual Gyrus-Middle Frontal Gyrus	-11 -57 2	-33 54 -6	p(FDR) < 0.001, T = 5.84, k = 286
Left Lingual Gyrus-Middle Frontal Gyrus	-11 -57 2	-27 42 9	Local maximum
Left Lingual Gyrus-Middle Frontal Gyrus	-11 -57 2	-36 42 -12	Local maximum
Left Lingual Gyrus-Cingulate Gyrus	-11 -57 2	6 -15 30	p(FDR) = 0.042, T = 4.75, k = 79

## Discussion

This study found that patients with schizophrenia in a stable phase of their illness showed increased functional connectivity compared with healthy controls between the left lingual gyrus, middle frontal gyrus, and the cingulate cortex. In contrast to previous studies, the brain regions linked to the DMN, the ECN, and the SN did not reveal any statistically significant differences between healthy controls and schizophrenia patients despite evidence for decreases in connectivity in the patients. Therefore, our hypothesis that lingual gyrus aberrant connectivity is a trait marker in schizophrenia was confirmed. However, we failed to confirm our hypothesis that aberrant connectivity in the brain areas linked to the three large scale brain networks reflects similar trait characteristics.

The lack of statistical significance in the reductions in connectivity in major neural networks in our sample of patients with schizophrenia could be attributed to the fact that our sample consisted of patients in a stable phase of their illness. One possibility is that functional dysconnectivity within large scale resting-state networks is state-related and disappears when symptoms subside and hence may not be a trait biomarker of schizophrenia. The other possibility is a type 2 error due to the small sample size.

Further support to this is provided by our finding of hyperconnectivity between the left lingual gyrus and the middle frontal gyrus, and the cingulate gyrus. A previous meta-analysis of resting-state brain activity reported bilateral lingual gyrus as the only region showing hyperactivity in schizophrenia (Kuhn & Gallinat, 2013). Wang, Rau, Li, Chen, and Yu, have reported higher connectivity between the lingual gyrus and thalamus in patients with schizophrenia (Wang et al., 2015). The finding of hyperconnectivity in our sample suggests that lingual gyrus hyperconnectivity may be a trait neuroimaging marker for schizophrenia and is consistent with the results of Kuhn and Gallinat, (2013) and Panaliyappan and Liddle, (2014).

Higher resting-state brain entropy in the lingual gyrus has been proposed to reflect the hyperprocessing of facial and vision signals noted in delusional hallucinatory schizophrenia symptoms (Xue et al., 2019). Wang et al. reported increased functional connectivity between the thalamus and the lingual gyrus, which could be related to sleep disturbances noted in schizophrenia (Wang et al., 2015).

Our results explain the divergent findings in the field of functional connectivity in schizophrenia. Even though Friston et al. suggest that the psychotic brain is disconnected and attributes the divergent findings of the field to that dysconnectivity, divergent findings can potentially be explained through studies using samples from patients in different stages of schizophrenia (Friston et al., 2016). Perhaps functional connectivity deficits brought about by the disorder dissipate when the psychotic brain is in remission. In contrast, other functional connectivity abnormalities that were pre-existing and indicative of risk for psychosis remain when patients are in a stable phase of their illness.

Previous research studying hyperconnectivity of the default mode network and the cognitive control network for differentiating treatment-resistant and treatment-sensitive suggest that medications such as antidepressants can normalise hyperconnectivity in the DMN (Dichter et al., 2015). It was further found that this normalisation only occurred in the posterior areas and not the anterior (Li et al., 2013). Other research has also suggested that prior to treatment, subcallosal cingulate gyrus, hyperconnectivity of the DMN and cognitive control network predict more significant clinical improvements after TMS suggesting the importance of hyperconnectivity of the networks (Liston et al., 2014). Further studies in this area have found hyperconnectivity between the thalamus and other brain regions in individuals with schizophrenia compared to a controlled group. Research into functional connectivity displayed hyperconnectivity between the thalamus and cortical regions, specifically in individuals with schizophrenia, suggesting that these abnormalities may relate to the underlying physiological mechanisms of impairment in schizophrenia (Yasuda et al., 2020).

Interestingly, our results were contained in the left lingual gyrus rather than bilaterally. Recent research conducted by Xue et al., (2019) found high levels of temporal brain entropy in the left lingual gyrus in patients with schizophrenia. The study suggested this was because the left lingual gyrus has been associated with heightened visual issues in schizophrenia as it distorts the processing of sensory stimuli, as supported by previous studies (Oertel et al., 2007). Other studies have suggested that such increases in the temporal brain entropy in the left lingual gyrus display the hyper-processing of visual signals, indicated as delusions and hallucinations in schizophrenia (Lee et al., 2002). The Xue et al. study also found increases in the left precuneus; previous studies have identified this as a vital area in the functions of the DMN (Raichle et al., 2001). Resting-state activity research in the left precuneus has identified the area as a facilitator for brain functions during tasks. Therefore such increases in the left precuneus may be due to the need to balance temporal brain entropy dysfunctions in other areas of the brain.

Our results contrast the findings of Manoliu et al. and Kasparek et al., who support that resting-state networks are disrupted even during psychotic remission (Kasparek et al., 2013; Manoliu et al., 2013). In particular, Manoliu et al. suggest that anterior insula FC dysfunction and its relationship to altered between-network interactions is present during psychotic remission and supports resting-state network reorganization during schizophrenia which persists once the acute phase of the disease has dissipated. Kasparek et al. suggest that abnormal FC of several brain networks is still present after an episode of schizophrenia is in remission and postulate such a finding could be a marker of disease stabilisation reflected by adaptive compensatory processes (Kasparek et al., 2013).

The differences between our results and the two studies could be explained by the sample size, preprocessing of fMRI images, or functional connectivity analysis methods. One of the studies had a smaller sample size compared to the current study (Manoliu et al., 2013); the other had a similar sample size as the current study (Kasparek et al., 2013). The second explanation for the different results could be attributed to the methods used for nuisance regression during preprocessing. Manoliu et al. accounted for motion by only excluding participants based on excessive head motion metrics (linear shift > 3 mm across runs and on a frame-to-frame basis, rotation > 1.5°), which led to none of their participants being excluded and did not account for motion during their analysis. They did not account for WM and CSF signals. Kasparek et al. accounted for motion using realignment and excluding components that had signal in WM and CSF that was unspecified (Kasparek et al., 2013).

Multimodal brain imaging data is extremely useful in clarifying functional and structural differences in individuals with schizophrenia and healthy controls (Yang et al., 2016). Such research is valuable as it capitalises on each imaging modality and their inter-relations in one analysis; such computational methods are beneficial for clinical research in understanding the underlying mechanisms of schizophrenia and its progression (Su et al., 2012). A combination of structural and functional data provides information on aberrant connectivity and changes in brain patterns thus increasing the confidence of the results. The useful part of including a multimodal imaging dataset is that different modalities add a new perspective on the brain analysis along with data fusion. There have been several advances in data fusion recently helping integrate large amounts of complex datasets efficiently (Sui et al., 2009).

The strengths of the present study include a well-characterised sample of patients with schizophrenia in a stable phase of their illness, a seed-based FC approach, and robust motion, WM and CSF signal nuisance regression. However, this study’s results should be cautiously approached due to some limitations, such as the small sample size potentially leading to a type 2 error. Most of the patients were on antipsychotic medication. Though we found no correlation between FC and dose of antipsychotic medication, it is not possible to rule out the effect of medication on FC. Also, the scans were 5 min long acquired with participants' eyes closed. Studies have found that the length of the resting-state fMRI scans impacts the reliability (Dijk et al., 2010). Research has suggested that only 50% reliability can be attained with a 5 min resting-state scan (Liao et al., 2013). Research studying the test–retest reliability of fMRI scans in a range of durations from 3 to 27 min suggested that the reliability was improved when the scans were 13 min long (Birn et al., 2013). The research also did not measure the level of arousal of the participants, which could have impacted the data acquired. Furthermore, the potential for our findings to be trait related is based on the stable status of our schizophrenia sample. Longitudinal designs are needed to firmly establish the trait potential of left lingual gyrus hyperconnectivity. Finally, our sample included patients with schizophrenia and schizoaffective disorder. The extent to which this comorbidity drives some of our results is unclear and should be investigated in larger samples which would allow for such comparisons.

In conclusion, this study used seed-based functional connectivity analyses to investigate dysconnectivity between brain areas linked to the major neural networks and the left lingual gyrus and the rest of the brain in patients with schizophrenia in a stable phase of their illness. Our findings suggest that the major resting-state networks appear deficient in stable patients with chronic schizophrenia, albeit not at a statistically significant level, suggesting that resting-state network deficiency in schizophrenia may be state-related and not a trait marker. The left lingual gyrus exhibited increased functional connectivity with two areas in the brain (middle frontal gyrus and cingulate gyrus). As our patient group was clinically stable, increased functional connectivity between the left lingual gyrus and the middle frontal and cingulate gyri could potentially be a trait neuroimaging marker for schizophrenia. Future studies should aim to elucidate whether lingual gyrus functional hyperconnectivity is genuinely a trait neuroimaging marker for schizophrenia by testing it in a larger sample of patients and testing its predictive utility in longitudinal cohorts with multivariate/multimodal datasets (Kandilarova et al., 2021).

## Supplementary Information

Below is the link to the electronic supplementary material.

Supplementary file1 (DOCX 142 KB)

## Data Availability

The datasets used or analysed during the current study are available from the corresponding author on reasonable request.
