# Supplementary material for: Trait related aberrant connectivity in clinically stable patients with schizophrenia: A seed based resting state fMRI study
Source: Brain Imaging Behav. 2022 Oct 14;16(6):2705–14. doi: 10.1007/s11682-022-00731-9 (PMC9712324; doi:10.1007/s11682-022-00731-9)

**Trait Related Aberrant Connectivity in Clinically Stable Patients with Schizophrenia: A Seed Based Resting State fMRI Study**

Paris Alexandros Lalousis, MSc^a,b*^, Aanya Malaviya^a,b^, BSc Rachel Upthegrove, MBBS FRCPsych, PhD^a,b^, Kareen Heinze, PhD^a,b^, Ana Diukova, PhD^c^, Dorothee Auer, MBBS, PhD^c^, Peter Liddle, MBBS, PhD^c^, & Pavan Mallikarjun, MBBS, PhD^a,b,c^

^a^Institute for Mental Health, University of Birmingham, Birmingham, B15 2SA, United Kingdom

^b^Centre for Human Brain Health, University of Birmingham, Birmingham, B15 2SA, United Kingdom

^c^School of Medicine, University of Nottingham, Nottingham, NG7 2RD, United Kingdom

^*^Corresponding author**:** Paris Alexandros Lalousis, BSc, MSc, School of Psychology, University of Birmingham, 52 Pritchatts Road, Birmingham, B15 2SA, United Kingdom ([pal532@student.bham.ac.uk](mailto:pal532@student.bham.ac.uk))

*Supplementary Methods and Results*

- 1. **Power analysis**

**
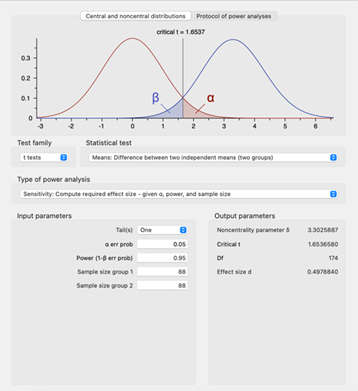

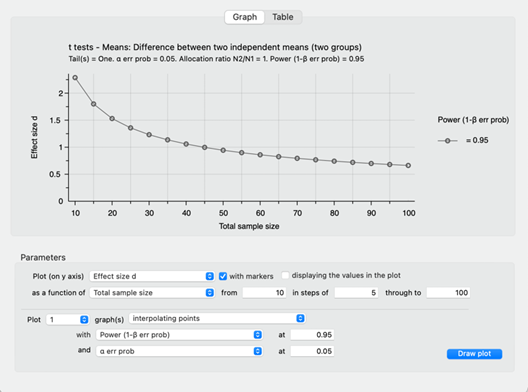
**

**1.2 Clinical Correlations**

| *Correlations* | | | | | | | |
| --- | --- | --- | --- | --- | --- | --- | --- |
|  | | Lingual_Gyrus_MFG | Lingual_Gyrus_Cingulate | Duration_of_Illness | GAF_Symptom_Severity | GAF_Level_of_Functioning | SSPI |
| Lingual_Gyrus_MFG | Pearson Correlation | 1 | .402^*^ | .244 | .129 | .156 | -.260 |
|  | Sig. (2-tailed) |  | .017 | .344 | .635 | .563 | .314 |
|  | N | 35 | 35 | 17 | 16 | 16 | 17 |
| Lingual_Gyrus_Cingulate | Pearson Correlation | .402^*^ | 1 | -.024 | -.018 | .064 | .127 |
|  | Sig. (2-tailed) | .017 |  | .928 | .948 | .813 | .627 |
|  | N | 35 | 35 | 17 | 16 | 16 | 17 |
| Duration_of_Illness | Pearson Correlation | .244 | -.024 | 1 | -.135 | -.096 | .343 |
|  | Sig. (2-tailed) | .344 | .928 |  | .618 | .723 | .178 |
|  | N | 17 | 17 | 17 | 16 | 16 | 17 |
| GAF_Symptom_Severity | Pearson Correlation | .129 | -.018 | -.135 | 1 | .968^**^ | -.728^**^ |
|  | Sig. (2-tailed) | .635 | .948 | .618 |  | <.001 | .001 |
|  | N | 16 | 16 | 16 | 16 | 16 | 16 |
| GAF_Level_of_Functioning | Pearson Correlation | .156 | .064 | -.096 | .968^**^ | 1 | -.687^**^ |
|  | Sig. (2-tailed) | .563 | .813 | .723 | <.001 |  | .003 |
|  | N | 16 | 16 | 16 | 16 | 16 | 16 |
| SSPI | Pearson Correlation | -.260 | .127 | .343 | -.728^**^ | -.687^**^ | 1 |
|  | Sig. (2-tailed) | .314 | .627 | .178 | .001 | .003 |  |
|  | N | 17 | 17 | 17 | 16 | 16 | 17 |
| *. Correlation is significant at the 0.05 level (2-tailed). | | | | | | | |
| **. Correlation is significant at the 0.01 level (2-tailed).  **1.3**  A two-sample T-Test was employed to assess if there was a difference in head motion between the two study groups (healthy controls and patients). The t-test results revealed no difference between the groups, t (33) = .082, p=.935. | | | | | | | |
| **1.4**  In order to assess whether GMV loss could explain some of our connectivity findings we extracted the mean raw GMV intensity values of the ROIs using MarsBar and then performed a correlation analysis to identify whether the FC results correlated with GMV values in the region. We found no significant correlations between FC and GMV. | | | | | | | |


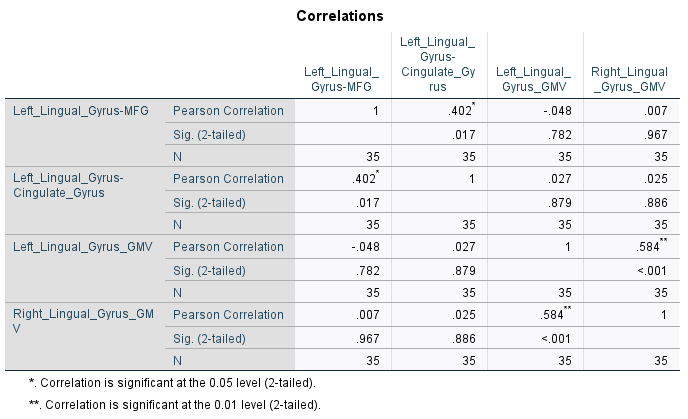

Supplement: Supplementary file 1 — Supplementary file1 (DOCX 142 KB) [file 11682_2022_731_MOESM1_ESM.docx]
